# The Impact of Microorganisms on the Performance of Linseed Oil and Tung Tree Oil Impregnated Composites Made of Hemp Shives and Corn Starch

**DOI:** 10.3390/microorganisms11020477

**Published:** 2023-02-14

**Authors:** Dovilė Vasiliauskienė, Giedrius Balčiūnas, Renata Boris, Agnė Kairytė, Jaunius Urbonavičius

**Affiliations:** 1 Department of Chemistry and Bioengineering, Vilnius Gediminas Technical University, Sauletekio av. 11, LT-10223 Vilnius, Lithuania; 2 Laboratory of Thermal Insulating Materials and Acoustics, Institute of Building Materials, Faculty of Civil Engineering, Vilnius Gediminas Technical University, Linkmenu st. 28, LT-08217 Vilnius, Lithuania; 3 Laboratory of Composite Materials, Institute of Building Materials, Faculty of Civil Engineering, Vilnius Gediminas Technical University, Linkmenu st. 28, LT-08217 Vilnius, Lithuania

**Keywords:** biocomposites, microorganisms, performance characteristics, biodegradation, biostability, enzymatic activity

## Abstract

In this study, the performance characteristics of hemp shives impregnated with linseed oil and tung tree oil (HS)- and corn starch (CS)-based biocomposites containing flame retardants were evaluated before and after treatment with the mixture of bacterium *Pseudomonas putida* and fungus *Rhizopus oryzae*. Enzymatic activities and physical-mechanical properties such as water absorption, thickness swelling, compressive strength, and thermal conductivity were tested to evaluate the suitability of selected composites for thermal insulation purposes. In addition, electron microscopy was used to investigate the impact of microorganisms on the microstructure of the material. It was determined that the type of oil used for impregnation significantly affects the properties of biocomposites after 6 months of incubation with mixture of bacterium *P. putida* and fungus *Rh. oryzae*. Biocomposites impregnated with linseed oil and after treatment with a mixture of microorganisms had cellulase activity of 25 U/mL, endo β-1-4-glucanase activity of 26 U/mL, lipase activity of 101 U/mL, only a 10% decrease in compressive strength, 50% higher short-term water absorption, unchanged swelling in thickness, and slightly decreased thermal conductivity compared to control biocomposites. At the same time, biocomposites with tung tree oil had a much more pronounced deterioration of the properties tested, cellulase activity of 28 U/mL, endo β-1-4-glucanase activity of 37 U/mL, lipase activity of 91 U/mL, two times lower compressive strength and two times higher short-term water absorption, 2.5 times greater thickness swelling, and a slightly increased thermal conductivity. We conclude that linseed oil provides better protection against the action of microorganisms compared to impregnation with tung tree oil.

## 1. Introduction

Currently, plant-based biocomposites are gaining a lot of attention from the scientific and industry communities. They are renewable and versatile building materials that are widely used in both indoor and outdoor environmental conditions. However, as plant-based materials, they are also susceptible to the negative impact of various microorganisms, for example, fungi and bacteria with accelerated growth under high moisture conditions [1,2]. To prolong the service life of wood, various technologies and methods are suggested, such as thermal modification or chemical processing and impregnation with various chemicals [3,4]. Wood preservatives such as creosote, pentachlorophenol, and copper are chemicals that are still used as biocides in application areas with limited contact with humans due to their toxicity and environmental concerns [5,6]. Therefore, the scientific community and industry seek alternative ways to replace existing toxic antifungal and antibacterial substances with preservatives with low environmental impact. Numerous natural options come from plant origin, for example, essential oils [7,8], extracts from plants [9], phenolics [10], and alkaloids [11] have been extensively studied as promising antifungal agents in wood protection. These studies show that green coatings for wood and even biocomposites are in high demand.

**Figure 1:**
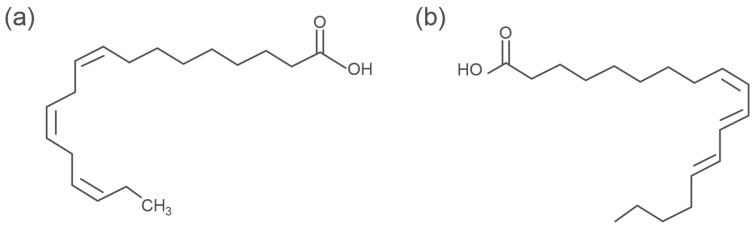
Conjugated and unconjugated double bonds of some components of drying oils: (**a**) linolenic acid in linseed oil; (**b**) α-eleostearic acid in tung tree oil.

Seed oils are also used as protective films to stop wood from rotting [12]. These oils have similar chemical structures because they consist of triglycerides along with unsaturated fatty acids, but there are some differences in the main composition and double bonds in each seed oil. For example, tung tree oil consists of approximately 82% α-eleostearic acid (Figure 1b), which has three conjugated double bonds. Conjugated double bonds results in an oil that cures faster compared to the oils with unconjugated double bonds, while linseed oil consists of approximately 53% linolenic acid with three unconjugated double bonds (Figure 1a), and soybean oil consists of approximately 58% linoleic acid having two unconjugated double bonds.

There are many studies that demonstrate the effectiveness of drying and semi-drying seed oils as substances for the formation of protective coatings on wood and biocomposites followed by cross-linking and solidification under the reaction with atmospheric oxygen. For example, tung tree oil-based impregnation was used for biobased particleboards from rice husk and soy protein. A noticeable improvement in water resistance, swelling in thickness, and mechanical performance was achieved [13]. The tung tree oil as a protective coating material can increase the stability of wood at higher temperatures [14]. In addition, it was previously shown [15] that the contact angle between a water drop and linseed oil impregnated wood, if sufficiently dried (up to 28 days of drying), may be even greater than for tung tree oil impregnation. Moreover, the same authors demonstrated that even better performance can be obtained by mixing the oils, obtaining a lower viscosity and an increased polymerization rate of the oil mixture. Although biobased water repellents for wood or biocomposites are desired, drying plant oils are a popular option. In these traditional applications, the tung tree oil film is biodegradable in the environment after the end of its service life.

However, the biodegradation process of the tung tree and linseed oil coatings remains an open case. Compared to the huge potential as protective coating materials for wood and biocomposites, specific knowledge is lacking, especially about the impact of the most commonly found fungi, such as *Rh. oryzae* and *A. fumigatus*, and bacteria such as *P. putida*. Therefore, the objective of the present study is to determine the impact of fungi and bacteria on performance characteristics such as density, thermal conductivity, water absorption, thickness, and compressive strength of hemp shives (HS)- and corn starch (CS)-based biocomposites impregnated with tung tree and linseed oils. The current study is a follow-up of previously published results [16], where the authors analyzed the effectiveness of linseed, tung tree, and hemp oils as protective coating materials for biocomposites based on HS and CS. Although the efficacy of drying oils was proved, it is of great importance to analyze the biodegradation aspect of such materials and test their performance characteristics to ascertain the stability of properties under fungal and/or bacterial attack.

## 2. Materials and Methods

### 2.1. Materials

For the development of biocomposites, HS with a fraction ranging from 2.5 to 5 mm was collected from the hemp fiber separation process in Lithuania. CS having a bulk density of 550 kg/m^3^ was used as a binder (Roquette, Lestrem, France). Flovan CGN-01 (FL) (Huntsmann Basel, Switzerland) and Nord-min 249 expandable graphite (EG) flakes (Nordmann, Rassmann GmbH, Hamburg, Germany) were used as liquid and solid flame retardants. Boiling water was used to activate the HS fibers. Linseed oil with a density of 0.929 ± 0.002 g/L was supplied by farmers in the Utena region, Lithuania, while tung tree oil with a density of 0.941 ± 0.002 g/L was purchased from JSC Pro colore, Lithuania, and used as a protective coating for biocomposites based on HS and CS.

For the incubation of biocomposites with microorganisms, the following materials were used: LB medium (liquid or solid with 15 g/L of agar, Formedium, Swaffham, UK), 10 g/L of NaCl (Carl Roth, Karlsruhe, Germany), 5 g/L of yeast extract (Sigma-Aldrich, Poznan, Poland) or PDA medium (VWR BDH Chemicals, Darmstadt, Germany) prepared using the manufacturer’s protocol. All media were autoclaved at 121 °C for 15 min.

### 2.2. Preparation of HS- and CS-Based Biocomposites and Their Incubation with Microorganisms

Biocomposites were formed from HS, CS, EG, FL, boiling water, linseed oil, and tung tree oil. To evaluate the impact of microorganisms on performance characteristics, biocomposites were prepared according to the compositions presented in Table 1.

Firstly, the 2.5–5 mm fraction HS was activated with boiling water, which defibrillates separate particles, thus assuring better adhesion with binding material. Further, activated HS particles were left to cool to an ambient temperature for 2 h and then drained for 10 min to remove excess water. Then, the scaled amounts of flame retardants, EG and FL, were added. The CS was sieved through a 0.63 mm mesh sieve into the prepared HS. The mixture obtained was thoroughly mixed for 3 min to achieve a homogeneous mass. Secondly, the mixture with HS and CS was poured into the metal mold with the size of 300 × 300 mm and compressed to 40% vol. To harden the prepared mixture, the metal mold was placed in a heating oven. The following steps of a heating mode were implemented: temperature increase to 160 ° C in 1 h at ~2.3 °C/min, temperature maintenance at 160 ° C for 6 h, temperature reduction to 20 °C in 1 h at ~2.3 °C/min. At the end of the hardening, the mold was left in the heating oven for a while and cooled to an ambient temperature. Hardened biocomposites were removed from the mold and impregnated with linseed and tung tree oils that were approximately from 43% to 94% of the total weight of the biocomposite. As biocomposites impregnated with linseed oil contained FL, a substance that increases the hydrophobicity of textile and fills the possible pores, the impregnated amount of linseed oil is lower compared to the one of tung tree oil. Impregnation was carried out in the vacuum desiccator for 5 min at 0.9–1 bar pressure. The vacuuming of the samples was repeated 10 times. The impregnated biocomposites were then hardened at a temperature of 90 °C in a ventilated drying oven.

Microorganisms were isolated from 5-year-old biocomposites according to the procedure described in study [17]. The bacteria that were used in this work are *P. putida*, OP247628, and the *Rh. oryzae* fungi, OP271776.

Bacterium *P. putida* was incubated in liquid LB medium, 10 g/L NaCl, 5 g/L yeast extract, at 30 °C temperature for 24 h. Then, a suspension of 10^9^ colony formation units (cfu)/mL was prepared in sterile 0.9% NaCl. The fungus *Rh. oryzae* was incubated in PDA medium from 10 to 21 days. After incubation, the spores were taken into 20 mL of sterile 0.9% NaCl and diluted to 10^6^ units/mL. Fungus *Rh. oryzae* and bacterium *P. putida* were mixed in equal parts. A total of 1 mL of the mixture was poured on HS- and CS-based biocomposites impregnated with linseed and tung tree oils. Incubation with microorganisms lasted 6 months.

### 2.3. Test Methods

#### 2.3.1. Enzyme Activity Assays

A preliminary qualitative assay of cellulase activities in solid media was performed for each identified bacteria and fungi separately, using Petri dishes with agar medium. For the identification of microorganisms that produce cellulase enzymes, they were placed in two sets of 1% carboxymethylcellulose medium (CMC,15 g/L agar (Formedium), 10 g/L carboxymethyl cellulose, 1 g/L NaNO_3_, 1.8 g/L, K_2_HPO_4_, 0.9 g/L MgSO_4_·6·H_2_O, 0.5 g/L KCl, 20 g/L (all Sigma-Aldrich). The inoculates of bacteria or fungi were described previously. Petri dishes with *P.putida* or *Rh. oryzae* inoculates were incubated from 3 to 28 days at 22 °C temperature. Cellulase activities were examined by determining transparent zones around colonies, which indicate cellulose hydrolysis after being flooded with 0.1% Congo red for 15 min and then 10 min with a 1 M NaCl solution. The cellulose was hydrolyzed by enzymes, so enzymatic index was calculated as the diameter of transparent zones divided by the diameter of microorganism colonies using the formula below:

(1) $$EI=\frac{Diameter\;of\;hydrolysis\;zone}{Diameter\;of\;colony}$$

where 12 samples were tested for each microorganism.

Enzymatic activities were measured for three samples of each composition in suspension with microorganisms after growth for 6 months. The suspension was prepared to measure cellulase activities using 0.25 g ± 0.05 g of biocomposite in 5 mL of 50 mM citrate buffer at pH 4.8. These samples were stirred at 22 °C temperature for one hour on an orbital agitator at 120 rpm, then centrifuged at 10,621 RCF for 10 min. Then, this suspension was filtered with a cellulose acetate membrane 0.45 µm, diam. 47 mm (Whatman, Cytiva, Meckenheim, Germany), using the vacuum system. Using this suspension, total cellulase activity was determined by the method recommended by IUPAC. Cellulase activity was measured using 500 μL of enzyme of suspension, and 50 mg filter paper in 1 mL of 50 mM citrate buffer at pH 4.8 as a substrate was used; incubation was performed for 2 h at 50 °C temperature. After this, 3,5-DNS acid (Alfa Aesar, Kandel, Germany ) was added and kept in boiling water for 10 min. The released glucose was measured at 540 nm using a microplate spectrophotometer (EON, Biotek, Winooski, VT, USA). Endoglucanase activity was measured using the same method as according to IUPAC recommendations, but using carboxymethylcellulose (CMC) as substrate with incubation for 1 h at 50 °C. The glucose released was assayed as previously described.

The suspension for the determination of lipase activities was prepared after 6 months of growth using three 0.5 g ± 0.05 g biocomposite samples of each composition in 20 mL 50 mM phosphate buffer of pH 7; all of this mixture was incubated in a water bath shaker (GFL) for 24 h at 120 rpm at 37 °C. After this, the supernatant was prepared as previously described. Lipase activity was determined using the colorimetric method described by Zhao et al. [18]. The substrate p-nitrophenyl palmitate (NPP, Alfa Aesar, Kandel, Germany) was converted after enzymatic treatment to p-nitrophenol (p-NP), which was measured by absorbance at 410 nm spectrophotometrically (EON, Biotek, Winooski, VT, USA).

#### 2.3.2. Structural and Physic-Mechanical Tests 

For microstructural analysis of HS- and CS-based biocomposites impregnated with different oils, scanning electron microscopy (SEM) was performed using JEOL SM–7600F (JEOL Ltd., Tokyo, Japan). The samples were sputter coated with a thin gold layer to proceed with imaging using the device QUORUM Q150R ES (Quorum Technologies Ltd., Lewes, UK).

The apparent density measurements were carried out according to EN 1602 [19] requirements, while the compressive strength values were obtained according to EN 826 with a Hounsfield test machine H10KS (Tinius Olsen Ltd., Surrey, UK). The test was carried out on three samples, with a size of 50 mm × 50 mm × 12 mm for each composition. Before the test was performed, all samples were kept for 6 h and the test was carried out under 23 ± 5 °C temperature conditions.

Compressive stress at 10% of deformation was measured based on the EN 826 [20] with testing machine H10KS (Hounsfield, Surrey, UK). Maximum loading force of the testing machine was 10 kN, with a loading speed accuracy of ± 0.05% and a loading accuracy of ± 0.5%. Before determination of the parameter, samples were maintained for not less than 6 h at (23 ± 5) °C temperature and (50 ± 5)% relative humidity conditions. Three samples with the size of 50 × 50 × 12 mm of each composition were used for the test.

Short-term water absorption by partial immersion was determined according to ISO 29767 [21], Method B. Four 200 mm × 200 mm samples for all compositions were used. All biocomposites were conditioned for ≥6 h at a temperature of 23 ± 5 °C prior to the test. The test was carried out for 24 h and the values were determined after draining the samples for 10 min on the draining stove.

The swelling of the thickness test was carried out according to EN 317 [22] requirements. The initial thickness of the biocomposite samples based on HS and CS was measured according to EN 325 [23]. The samples were immersed in the water. The bottom surface of all biocomposite samples was filled with water to a height of 25 ± 5 mm. The excess water was drained, the thickness of the samples was measured, and the respective values were calculated. All samples were kept at 20 ± 2 °C temperature and 65 ± 5% relative humidity before the test, until a constant weight was achieved. Three biocomposite samples with a size of 50 × 50 × 12 mm were used for the test.

Thermal conductivity was measured for three 300 mm × 300 mm × 12 mm samples based on EN 12667 [24] with a FOX 304 heat flow meter (TA Instruments, Eden Prairie, MN, United States of America). The direction of heat flow used during the test was upward. Average temperature during the test was 10 °C, and the temperature difference between the cold and hot plates was 20 °C.

In order to perform a statistical interpretation of the results, the Statsoft 8.0, 2007 software tool was implemented. The error values for interval prediction were calculated according to the following equation:

(2) $$Y_{i}^{pred.}=\bar{Y_{i}}\pm \delta$$

(3) $$\delta =t_{\alpha }\cdot S_{r}$$

where $t_{\alpha }$– Student criterion selected according to degree of freedom, which is calculated from f=m−n with the confidence level of 95%.

## 3. Results and Discussion

### 3.1. Enzymatic Activities and Microstructure of HS- and CS-Based Biocomposites That Were Impregnated with Different Oils

**Figure 2:**
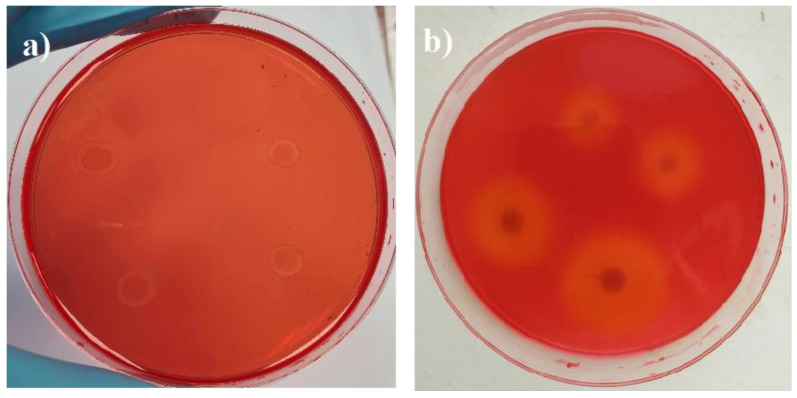
Visual assessment of cellulase activity in CMC medium by flooding Petri dishes with Congo red: (**a**) *P. putida* after growing for 13 days; (**b**) *Rh. oryzae* after growing for 12 days.

Cellulase activities were checked by measuring the zones around bacterium *P.putida* or fungus *Rh.oryzae* colonies that show cellulose hydrolysis. The addition of Congo red solution reveals hydrolyzed zones relative to the color contrast of the non-hydrolyzed medium. It is accepted that the diameter of clear zone establishes the bacteria’s capability to hydrolyze cellulose [25,26]. Therefore, Figure 2 presents the activity of cellulase in the CMC medium.

The Halo zones showed that the calculated enzymatic index of bacterium *P. putida* after 13 days was 1.2 ± 0.05, while for fungus *Rh. oryzae* it was 4 ± 0.5. The plate with *Rh. oryzae* had clearer zones compared to the bacterium *P. putida* because this fungus is a more active decomposer of cellulose than bacteria [27,28].

**Figure 3:**
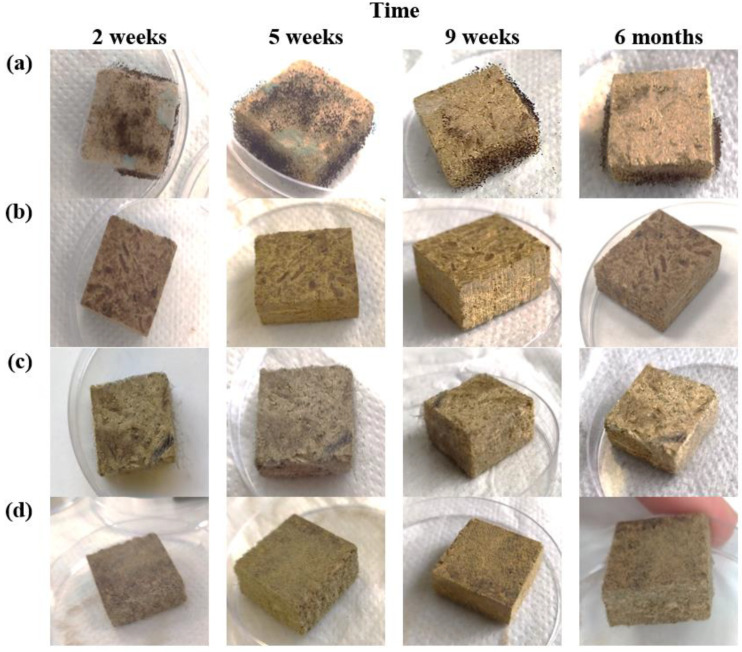
The surface appearance of HS- and CS-based biocomposites impregnated with different oils after 2 weeks, 5 weeks, 9 weeks, and 6 months: (**a**) control samples without linseed oil; (**b**) samples with linseed oil; (**c**) control samples without tung tree oil; (**d**) samples with tung tree oil.

Visually, it was established that there is no growth of both the bacterium *P. putida* and the mold fungus *Rh. oryzae* on the surface of biocomposite based on HS and CS with linseed oil (Figure 3). However, in HS- and CS-based biocomposite with tung tree oil, microorganism growth was visually detected only in the 6th month, while fungus growth at the 4th week for textile-based composite thermal insulation material was detected using the microscope [29]. Since some aerial hyphae and mycelium are visible, it can be stated that these are not bacteria, but fungi. When the suspension of fungus *Rh. Oryzae* and bacterium *P. putida* is applied, the growth of aerial hyphae and the mycelium of the fungus are visible.

**Figure 4:**
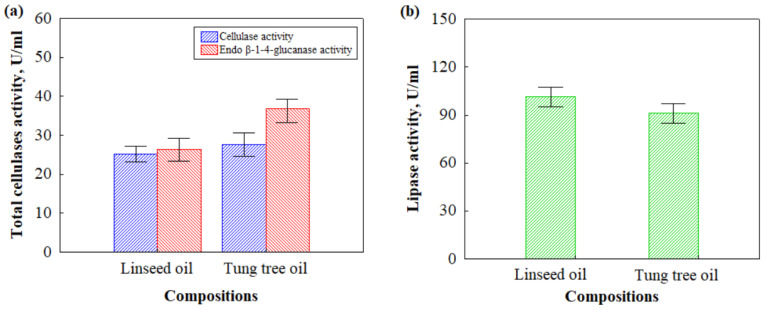
Enzymatic activities of HS- and CS-based biocomposites impregnated with different oils after incubation with microorganisms for 6 months: (**a**) cellulase and endo β-1-4-glucanase activities; (**b**) lipase activity.

During production of biocomposites, the HS were treated with boiling water, which activated the fibers on the surface of the HS particles. This process defibrillates HS particles, exposing their surface, which is then susceptible to attack by fungi. Furthermore, the combination of boiling water treatment and hot-pressing processes could expose polysaccharide components, hemicellulose, or cellulose segments, which are nutrients for fungus *Rh. oryzae* [30]. However, even though biocomposites based on HS and CS impregnated with linseed and tung tree oils were produced using the same technological scheme, the difference in cellulase and lipase activities (Figure 4) can still be observed. Additionally, no amylase activity was found for both biocomposite compositions with different oils, indicating the effectiveness of the oil films formed to protect the CS used as a binding material in biocomposites. However, total cellulase activity showed that cellulase activity and endo β-1-4-glucanase activity of HS- and CS-based biocomposites impregnated with linseed oil are, respectively, 8% and 40% lower compared to samples impregnated with tung tree oil (Figure 4a). However, the difference between cellulase activities for biocomposites impregnated with linseed oil and tung tree oil was insignificant. Furthermore, biocomposites impregnated with linseed oil had 10% higher lipase activity compared to biocomposites impregnated with tung tree oil.

It was concluded long ago [31] that certain plant oils, such as linseed oil and tung tree oil, are able to reduce the amount of blue mold in tobacco caused by *Peronospora tabacina* due to the presence of linolenic acid or α-eleostearic acid, respectively. Furthermore, a new study [32] showed the possible efficiency of linseed oil and tung tree oil as protective coatings of 53 to 100% and 32 to 100%, respectively, to control oil-impregnated bean leaves inoculated with *Uromyces appendiculatus*. However, the importance of impregnation time before the inoculation process was also shown, because the efficacy of protective coatings is reduced over time, as demonstrated in Figure 3.

Fungi have the ability to deteriorate cell walls in wood and wood-based products, as well as interact with other bacteria and fungi to decompose organic substances such as cellulose, hemicellulose, lignin, and other specific components in wood or wood-based products to cause microstructure alterations [33,34].

**Figure 5:**
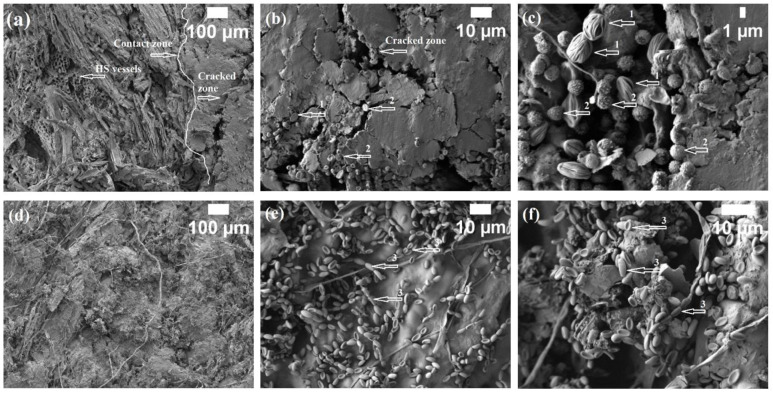
Microstructure of HS- and CS-based biocomposites impregnated with different oils after incubation for 6 months (1—fungus *Rh. oryzae*, 2—fungus *A. fumigatus*, 3—bacterium *P. putida*): (**a**) surface of biocomposite with linseed oil (magnification ×100); (**b**) surface of biocomposite with linseed oil (magnification ×1000); (**c**) 5 mm depth of biocomposite with linseed oil (magnification ×3000); (**d**) surface of biocomposite with tung tree oil (magnification ×100); (**e**) surface of biocomposite with tung tree oil (magnification ×1000); (**f**) 5 mm depth of biocomposite with tung tree oil (magnification ×1500).

Therefore, to further explore the growth of fungus hyphae and bacteria in biocomposites impregnated with different oils, the infected samples were dissected, and microscopic morphology was observed. SEM images (Figure 5) of oil-impregnated HS- and CS-based biocomposites revealed that *Rh. oryzae* and *A. fumigatus* fungi dominate on the surface and in deeper layers of HS- and CS-based biocomposites (Figure 5c). Although the suspension of bacterium *P. putida* and fungus *Rh. oryzae* was used to incubate biocomposites, fungus *A. fumigatus* can be detected as well. Although all samples were sterilized under UV light prior to incubation with microorganisms, fungus *A. fumigatus* recovered under sufficient thermal and humidity conditions. Furthermore, it should be mentioned that biocomposites impregnated with linseed oil contain the Flovan CGN-01 flame retardant in their composition (Table 1). This flame retardant is slightly acidic and has a pH value of 4.5, but its exact composition is not publicly available, even though it is known that it has properties of Lewis acids. The more acidic environment could be the cause of two types of fungi in HS- and CS-based biocomposites impregnated with linseed oil [35]. It can also be seen in Figure 5a that the contact zones between HS and CS linseed oil were partially destroyed by fungi, which revealed the vessels of HS; in Figure 5b, the layer of CS and the layer of linseed oil are cracked, and this gives a path for the fungi to penetrate further into deeper layers of HS and CS.

Figure 5d–f reveals the microstructure of biocomposites based on HS and CS impregnated with tung tree oil. It can be clearly seen that the dominant microorganism in this composition is the bacterium *P. putida*. Interestingly, this bacterium was found only in biocomposite compositions impregnated with tung tree oil, although previously [36] it was found that linseed oil-impregnated wood had initial antimicrobial efficacy that was greater than for wood without impregnation. Furthermore, the antifungal efficacy of tung tree oil was proven in a previous study [37], which tested the resistance of spruce and beech wood to fungal attack. Tung tree oil is more effective compared to linseed oil because it has predominantly conjugated double bonds, that is, more than 80% α-eleostearic acid, which allows this oil to make more resonance radicals and simplify polymerization and cross-linking that leads to hydrophobic layer formation to inhibit fungal growth (Figure 5f) [38] but not robustness to bacteria growth [39]. At the same time, the effect of the flame retardants on the microbial growth is less evident. Since the exact composition of Flovan CGN-01 is not publicly available, it is difficult to judge its impact on the growth of the microorganisms tested. However, it was demonstrated using Next Generation Sequencing (NGS) analysis that the addition of Flovan CGN-01 into the biocomposites changes the composition of microbiota growth on the unprotected board samples compared to the ones without the flame retardants. A similar effect was observed for the expandable graphite (EG) flakes as the flame retardant [17]. It was previously demonstrated that different carbon nanomaterials such as nanotubes and graphene/graphene oxide exhibit antimicrobial properties [40]. Further investigations of the antimicrobial activities of flame retardants used in this work are currently under way. 

### 3.2. Density and Thermal Conductivity of Biocomposites before and after Impact of Microorganisms

It is well known that the density of building materials is the main parameter that determines various properties for a selected application. Its numerical value depends mostly on the production technology used and the raw materials. For example, using similar technological parameters, Anosike-Francis et al. [41] obtained wood chips and recycled polypropylene-based composites with an average density ranging from 1047 kg/m^3^ to 1137 kg/m^3^, while Martinez-Lopez et al. [42] used wood sawdust and thermoplastic polyethylene terephthalate to obtain boards with a density range of 1050–1100 kg/m^3^.

**Figure 6:**
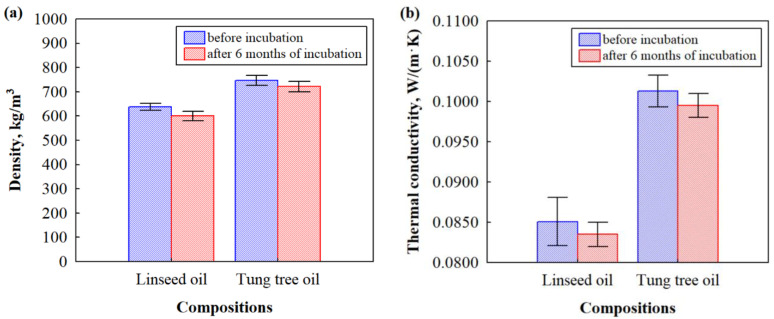
The impact of microorganisms on the selected properties of HS- and CS-based biocomposites impregnated with different oils before and after incubation for 6 months: (**a**) density; (**b**) thermal conductivity.

However, the biocomposites based on HS and CS analyzed before incubation with microorganisms had slightly lower values—an average density of 638 kg/m^3^ for biocomposite impregnated with linseed oil and 747 kg/m^3^ for biocomposite impregnated with tung tree oil (Figure 6a).

However, after incubation of biocomposites based on HS and CS with a mixture of bacterium *P. putida* and fungus *Rh. oryzae* for 6 months, it can be observed that the density was reduced by 6% for biocomposite impregnated with linseed oil and by 3% for biocomposite impregnated with tung tree oil. As our previous study showed [16], tung tree oil cross-links more densely and dries more rapidly to form a thicker film in the structure of biocomposite due to a higher number of carbon–carbon double bonds. According to microstructural analysis, the tung tree oil film on the surface of HS has a thickness of ~1 μm, while for linseed oil it is ~0.6 μm. It means that tung tree oil fills the pores and voids in the obtained biocomposite, as well as the valves, vessels, and tracheids in HS, consequently reducing the possibility for fungal hyphae to penetrate. Although, as Figure 3 and Figure 5d–f show, it still occurs after 6 months of incubation with microorganisms because the time for fungus growth test plays an important role because fungus infestation risk increases with incubation time [43,44] as the weight loss of the samples increases. It can also be noted that the composition of biocomposite impregnated with tung tree oil includes expandable graphite as a flame retardant, which is a carbon-based material that reduces susceptibility to microbial degradation of composite materials [45]. This is expected because the longer the fungus exposure time, the more degradation of lignocellulose materials occurred. Additionally, as density and microstructure changes in HS- and CS-based biocomposites impregnated with different oils after incubation with microorganisms, other characteristics, such as thermal conductivity, are altered as well. In Figure 6b, it can be seen that the thermal conductivity of biocomposites based on HS and CS and impregnated with linseed oil decreased by almost 1.9%, while for biocomposites impregnated with tung tree oil it decreased by 1.7%. As the density of the obtained biocomposites decreased slightly, the thermal conductivity decreased as well. The damage caused by fungus and bacterium to the structure of all biocomposites resulted in disintegrated contact zones between HS and CS, thus opening the pores in HS and creating the air gaps. As the air is a good thermal insulator, it reduces the overall thermal conductivity value of microorganism-impacted biocomposites.

### 3.3. Water Absorption and Compressive Strength Properties of Biocomposites before and after the Impact of Microorganisms

**Figure 7:**
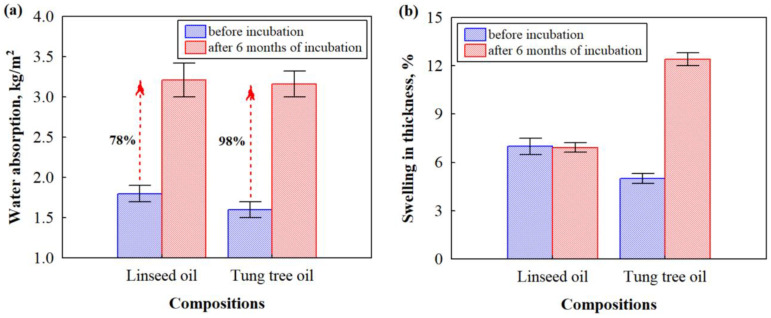
Moisture-related properties of HS- and CS-based biocomposites impregnated with different oils before and after incubation for 6 months: (**a**) short-term water absorption after immersion in water for 24 h; (**b**) swelling in thickness after immersion in water for 24 h.

Constant water absorption and/or desorption can destroy the appearance and long-term potency of wood or wood-based products, therefore, one of the requirements for outdoor and indoor applications is to test water resistance. Figure 7 shows short-term water absorption and swelling in thickness graphs of biocomposites based on HS and CS impregnated with linseed and tung tree oils before and after incubation with microorganism. The impregnation of biocomposites with linseed oil leads to slightly higher water absorption values compared to the impregnated biocomposites with tung tree oil. The average value of the parameter for biocomposites with linseed oil was 1.8 kg/m^2^, while for biocomposites with tung tree oil the value reached 1.6 kg/m^2^. The results obtained are highly consistent with the previous study [12], where the contact angles of the water droplets tested for wood were 107.2° for tung tree oil and 97.8° for linseed oil, indicating a higher efficacy of tung tree oil as a water-resistant coating. Moreover, composition biocomposites with tung tree oil contain EG as a flame retardant, which protects from water penetration into the deeper layers of biocomposites by filling some of the voids, cracks, and gaps formed between HS and CS. The tendency to swelling in the thickness parameter of HS- and CS-based biocomposites impregnated with different oils is the same as for water absorption. The composition of biocomposites impregnated with linseed oil had an average swelling thickness value equal to 7%, while the biocomposites impregnated with tung tree oil were 5%. Similar tendencies were observed by [13] in rice husk and particle boards based on soybean proteins impregnated with tung oil, and by [46] for wax emulsion-impregnated wood. In both studies, the partial coating of the porous sites caused a significant reduction in the rate of water absorption and swelling results.

However, exposure of the HS- and CS-based biocomposite samples impregnated with different oils leads to increased water absorption and swelling in thickness compared to samples prior to incubation with microorganisms. After incubation with microorganisms for 6 months, water absorption of biocomposites impregnated with linseed oil deteriorated by 78% and by 98% for biocomposites impregnated with tung tree oil, while thickness swelling remained the same for biocomposites impregnated with linseed oil and increased by almost 40% for biocomposites impregnated with tung tree oil.

**Figure 8:**
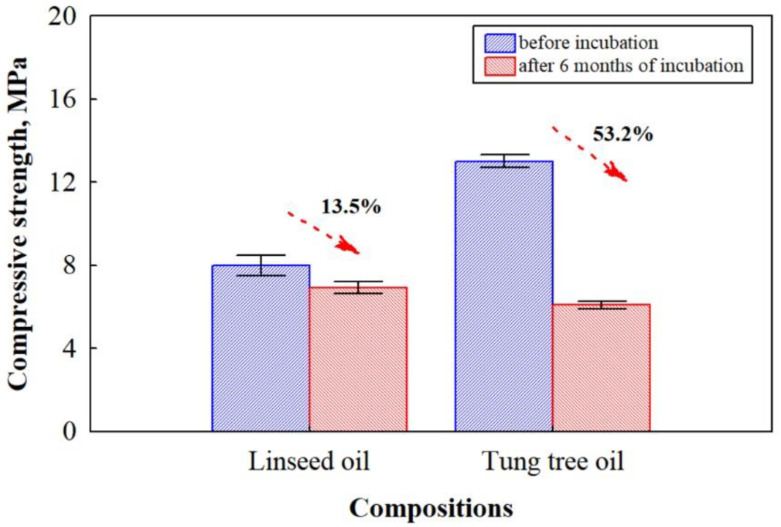
Compressive strength of biocomposites based on HS and CS and impregnated with different oils before and after 6 months of incubation.

This could be explained by the fact that the fungus degrades lignocellulosic materials and results in the formation of pores, cavities, and cracks in the hardened CS, which propagates the penetration of water molecules into deeper layers of biocomposites, thus increasing water uptake. These results are in great agreement with previous studies [47]. The reason why the relative increase in water absorption and swelling of the thickness was higher for biocomposites impregnated with tung tree oil can be explained by the previous results of its total cellulase activity (Figure 4), indicating a higher rate of HS decomposition. Interestingly, biocomposites impregnated with linseed oil had unchanged swelling of the thickness. It can be assumed that linseed oil has an antifungal potency because of the abundance in α-linolenic and linoleic acids reducing the ability of fungus to cause the breakage of contact zones between HS and CS. Another important characteristic of composite materials is compressive strength. The results of compressive strength performed on HS- and CS-based biocomposites impregnated with different oils before and 6 months after incubation with microorganisms are shown in Figure 8.

The results indicate that the strength characteristic of biocomposites impregnated with tung tree oil before incubation with microorganisms is 63% greater compared to biocomposites impregnated with linseed oil. It is suggested that a synergistic effect of tung tree oil and CS in biocomposites plays a crucial role due to the hydrogen bond between the free carboxylic groups in tung tree oil and the hydroxy groups in CS [48]. However, the compressive strength values of HS- and CS-based biocomposites deteriorate significantly after 6 months of incubation with microorganisms. The parameter reduces by 13.5% for biocomposites impregnated with linseed oil and by 53.2% for biocomposites impregnated with tung tree oil. It is assumed to be caused by the weak interfacial adhesion between the HS and the CS matrix due to the degraded lignocellulosic components under the fungal attack, as the strength characteristics of wood-based biocomposites are dependent on the interaction between the interface and the properties of the raw materials used [49].

## 4. Conclusions

The current study showed that microorganisms build colonies in deeper layers and on the surface of HS- and CS-based biocomposites impregnated with different oils 6 months after incubation with suspension of microorganisms. The dominant microorganisms in biocomposites impregnated with linseed oil were fungi *Rh. oryzae* and *A. fumigatus,* while, when investigating the biocomposites that were impregnated with tung tree oil, growth of bacterium *P. putida* was observed. The highest enzymatic activities were found in biocomposites impregnated with tung tree oil, and they were ~1.5 times higher compared to biocomposites impregnated with linseed oil 6 months after incubation. Structural analysis indicated that biocomposites impregnated with different oils had negatively affected the structure composition, were severely affected, and had disintegrated structure with CS destruction signs and unveiled HS vessels, which opens up a path of easier penetration for fungus into deeper layers of biocomposites impregnated with linseed oil. The damaged structure led to an alteration of the performance of HS- and CS-based biocomposites impregnated with different oils. The density was reduced by 6% for biocomposites impregnated with linseed oil and by 3.3% for biocomposites impregnated with tung tree oil, while the thermal conductivity values decreased by 1.9% and 1.7%, respectively. Furthermore, water absorption for biocomposites impregnated with linseed and tung tree oils was 78% and 98% higher, respectively, while thickness swelling remained unchanged for biocomposites impregnated with linseed oil and increased by 148% for biocomposites impregnated with tung tree oil. The highest drop in compressive strength was observed for biocomposites with tung tree oil coating, and it was 53.2% lower compared to biocomposites before incubation with microorganisms. Although tung tree oil showed exceptional behavior in biocomposites before incubation with microorganisms, it did not withstand the degradation ability of the microorganism solution used for the current study.

## Figures and Tables

**Table 1:** Compositions of HS- and CS-based biocomposites.

Raw Materials	Biocomposite With Linseed Oil	Biocomposite with Tung Tree Oil
HS, g	300	
CS, g	30	
Boiling water, l	2	
EG wt.% by CS mass	0	20
FL, g/L	30	0
Linseed oil, % by biocomposite mass	63.4	0
Tung tree oil, % by biocomposite mass	0	85.5

Note: HS—hemp shives; CS—corn starch; EG—expandable graphite; FL—Flovan CGN-01 flame retardant.

## Data Availability

Not applicable.
